# Predictive Performance of Oocyte Count for Clinical Pregnancy in GnRH Antagonist IVF Cycles: A Multivariable Analysis of 1171 Fresh Embryo Transfers over a 14-Year Period

**DOI:** 10.3390/medicina62061110

**Published:** 2026-06-07

**Authors:** Ömer Osman Eroğlu, Runa Özelçi, Ramazan Erda Pay, Cansın Eroğlu

**Affiliations:** Department of Obstetrics and Gynecology, Ankara Etlik Zübeyde Hanım Women’s Health Training and Research Hospital, Ankara 06010, Turkey; runakara@gmail.com (R.Ö.); drramazanpay@gmail.com (R.E.P.); cansin-yildiz@hotmail.com (C.E.)

**Keywords:** GnRH antagonist, in vitro fertilization, oocyte count, clinical pregnancy, multivariable analysis, ROC analysis, controlled ovarian stimulation

## Abstract

*Background and Objectives:* The optimal oocyte yield in gonadotropin-releasing hormone (GnRH) antagonist in vitro fertilization (IVF) cycles remains debated, and data specific to antagonist protocols are limited. This study evaluated the discriminative and independent predictive performance of oocyte count for clinical pregnancy in GnRH antagonist IVF cycles. *Materials and Methods:* This retrospective cohort included 1171 women undergoing their first GnRH antagonist IVF cycle with fresh embryo transfer at a single tertiary center (September 2007–December 2021). The primary outcome was an institutional composite pregnancy outcome (sustained β-hCG positivity with subsequent ongoing intrauterine pregnancy or live birth; biochemical and ectopic pregnancies were negative). Patients were grouped by oocytes retrieved (1–5, 6–10, 11–15, ≥16). Performance was assessed with logistic regression, ROC with 2000-iteration bootstrap, integrated discrimination improvement (IDI), continuous net reclassification improvement (NRI), and restricted cubic spline. Predefined subgroup analyses by age, regimen, and antral follicle count tertile were performed. *Results:* A positive outcome occurred in 430 patients (36.7%). After adjustment, oocyte count was not an independent predictor (adjusted odds ratio 0.999, 95% CI 0.979–1.020; *p* = 0.96). The full model (AUC 0.564, 95% CI 0.529–0.598) did not outperform oocyte count alone (AUC 0.532; bootstrap *p* = 0.11). IDI (0.011) and NRI (0.135) were statistically detectable but clinically trivial. Spline regression showed no non-linearity (*p* = 0.47). Findings were consistent across subgroups, and the narrow confidence interval excluded per-oocyte effects ≥1.10. *Conclusions:* In GnRH antagonist IVF cycles, oocyte count showed weak discriminatory performance and was not independently associated with fresh-cycle pregnancy. Oocyte yield should be interpreted alongside—rather than as a substitute for—established parameters such as age and ovarian reserve. The principal clinical value of a higher oocyte response may lie in cumulative rather than fresh-cycle success. Live-birth outcomes were not available, and the source institution was permanently closed in 2025; these limitations define the boundary of inference.

## 1. Introduction

Infertility affects approximately 15% of couples of reproductive age worldwide and represents a substantial public health concern [1]. In vitro fertilization (IVF) with embryo transfer has become one of the most effective therapeutic options for these couples, with contemporary pregnancy rates of 40–45% and live-birth rates of 30–35% per cycle [2,3].

Controlled ovarian stimulation (COS) is a cornerstone of IVF success. The goal is to promote multifollicular development in order to obtain a sufficient number of mature oocytes and maximize the likelihood of selecting high-quality embryos. Gonadotropin-releasing hormone (GnRH) antagonist protocols have become the most widely used COS strategy and are now recommended as a first-line approach in most IVF patients, owing to their shorter treatment duration, patient-friendly administration profile, lower risk of ovarian hyperstimulation syndrome (OHSS), and comparable live-birth rates relative to long GnRH agonist protocols [4,5,6]. Recent European Society of Human Reproduction and Embryology (ESHRE) guidelines formally endorse GnRH antagonist stimulation as the preferred first-line protocol [6].

Despite the wide adoption of antagonist protocols, the optimal number of oocytes to be retrieved remains debated. In a landmark analysis of 400,135 treatment cycles, Sunkara and colleagues reported that live-birth rates increased up to approximately 15 oocytes and plateaued thereafter [7]. Ji et al. suggested an optimal yield of 6–15 oocytes [8], while Van der Gaast et al. proposed 13 oocytes as the inflection point for fresh transfer success [9]. Against these findings, several studies have reported that supraphysiological estradiol concentrations during high-response COS may impair endometrial receptivity and compromise oocyte competence, arguing against the maximization of oocyte yield [10,11]. Milder stimulation strategies have been associated with reduced embryonic aneuploidy in randomized settings [12]. More recently, the POSEIDON concept has reframed the field from a binary poor/good responder classification toward a four-group low-prognosis stratification, emphasizing individualization of stimulation and interpretation of oocyte yield in the context of patient age and ovarian reserve [13,14]. Population-level analyses incorporating the POSEIDON framework have documented that cumulative live-birth rates track oocyte yield strongly when both fresh and frozen transfers are considered, but that fresh-cycle pregnancy rates plateau or decline at very high yields [15]. A broad evidence base has accumulated on clinical predictors of IVF outcome—including female age, ovarian reserve markers, and cycle-level variables such as oocyte and embryo yield—with systematic reviews synthesizing their relative prognostic weights and identifying oocyte number as one of the significant cycle-level predictors of pregnancy, although of smaller magnitude than maternal age [16]. Importantly, the majority of published analyses either pool mixed stimulation protocols or are derived predominantly from long agonist cohorts; data specific to GnRH antagonist stimulation remain comparatively limited [7,8,9,17]. Whether the number of oocytes retrieved is an independent and clinically useful predictor of pregnancy in GnRH antagonist cycles—and whether its predictive performance is robust after accounting for age, ovarian reserve, and cycle characteristics—has not been adequately addressed using contemporary analytic tools. The present study therefore evaluated, in a single-center cohort of 1171 women undergoing their first antagonist IVF cycle with fresh embryo transfer: (i) the association between oocyte yield and a positive pregnancy outcome; (ii) the independent predictive performance of oocyte count using multivariable logistic regression; (iii) the shape of the oocyte–pregnancy relationship through restricted cubic spline regression; and (iv) the consistency of these findings across predefined age, stimulation regimen, and ovarian reserve subgroups, together with reclassification-based analyses and a precision analysis of the null estimate.

## 2. Materials and Methods

### 2.1. Study Design and Setting

This retrospective cohort study was conducted at the Assisted Reproduction Unit of Ankara Etlik Zübeyde Hanım Women’s Health Training and Research Hospital (Ankara, Turkey), which operated as a tertiary referral center for assisted reproduction during the study period. All patient data, stimulation cycles, and clinical outcomes analyzed in this manuscript were accrued at that institution between September 2007—corresponding to the inception of the unit’s assisted reproduction services—and December 2021, which corresponds to the date at which the present study was conceptually designed. The ethics application defined, a priori, that the cohort would comprise all eligible patients from the inception of the unit’s ART services through the study design date, rather than a sampled time frame. The institution was permanently closed in 2025, and its clinical services are no longer operational; the present authors have since relocated to Ankara Etlik City Hospital. The dataset analyzed in this study represents the complete institutional record retrieved prior to closure, and no further retrospective data extraction from the original electronic medical record system has been possible since that date. The study was approved by the Institutional Ethics Committee of Health Sciences University Ankara Etlik Zübeyde Hanım Women’s Health Training and Research Hospital (Decision No. 2022/82, dated 8 June 2022) and was conducted in accordance with the Declaration of Helsinki. Owing to the retrospective nature of the study, the requirement for individual informed consent was waived.

### 2.2. Participants and Eligibility Criteria

Eligible patients were women aged 20–45 years undergoing their first IVF cycle with a GnRH antagonist protocol, in whom at least one oocyte was retrieved and a fresh embryo transfer was performed. Exclusion criteria were: age below 20 or above 45 years, use of a GnRH agonist protocol, second or subsequent IVF cycle, failure to retrieve any oocyte, cycle cancellation due to OHSS risk, total fertilization failure, suboptimal embryo development precluding transfer, and freeze-all cycles. After applying these criteria, 1171 consecutive patients—representing the complete eligible cohort of the unit from its ART inception through December 2021—were included in the analysis. This manuscript is reported in accordance with the STROBE guidelines for observational studies [18].

### 2.3. Stimulation Protocol and Laboratory Procedures

All patients received a GnRH antagonist protocol. Ovarian stimulation was initiated on menstrual cycle day 2 or 3 with recombinant follicle-stimulating hormone (rFSH)—either follitropin alfa (Gonal-f^®^, Merck Serono, Geneva, Switzerland) or follitropin beta (Puregon^®^, MSD/Organon, Oss, The Netherlands)—and/or human menopausal gonadotropin (hMG; menotropin, Menopur^®^, Ferring Pharmaceuticals, Saint-Prex, Switzerland); in a small subset of cycles, recombinant luteinizing hormone (rLH; lutropin alfa, Luveris^®^, Merck Serono, Geneva, Switzerland) was additionally co-administered. The starting dose (range 150–300 IU/day) was individually adjusted for age, body mass index (BMI), and ovarian reserve (antral follicle count [AFC] and, when available, anti-Müllerian hormone [AMH]). In patients with prior ovulation induction or intrauterine insemination cycles, the documented ovarian response during these preceding non-IVF treatment cycles was additionally considered in selecting the starting gonadotropin dose; for treatment-naïve patients, the starting dose was determined on the basis of baseline characteristics alone. The GnRH antagonist—either cetrorelix (Cetrotide^®^, Merck Serono, Geneva, Switzerland) or ganirelix (Orgalutran^®^, MSD/Organon, Oss, The Netherlands), 0.25 mg/day subcutaneously—was initiated when the leading follicle reached 12–14 mm and continued until the day of trigger. Final oocyte maturation was induced with either recombinant human chorionic gonadotropin (rhCG; choriogonadotropin alfa, Ovitrelle^®^, Merck Serono, Geneva, Switzerland; 250 μg) or urinary hCG (uhCG; Pregnyl^®^, MSD/Organon, Oss, The Netherlands; 5000–10,000 IU) once at least two follicles had reached ≥17 mm. The specific trigger preparation administered for each cycle (rhCG vs. uhCG) was not retained as a separate categorical variable in the longitudinal institutional database during the study period and could therefore not be incorporated as a covariate in the multivariable analysis or stratified across in subgroup comparisons; the two preparations have been shown to yield comparable ongoing-pregnancy and live-birth rates in a Cochrane systematic review and meta-analysis of randomized trials [19]. Oocyte retrieval was performed under transvaginal ultrasound guidance 34–36 h after trigger. Intracytoplasmic sperm injection was performed in all cycles, reflecting unit-wide practice during the study period. Embryo transfer was carried out under ultrasound guidance on day 2, 3, or 5 after retrieval. The specific embryo transfer day for each cycle (cleavage-stage, day 2 or 3, vs. blastocyst-stage, day 5) was not retained as a separate categorical variable in the longitudinal institutional database during the study period and could therefore not be incorporated as a covariate in the multivariable analysis or stratified across in subgroup comparisons; the implications of this database constraint for residual confounding are discussed in Section 4.1. Luteal-phase support was provided with vaginal progesterone.

### 2.4. Outcome Definition and Grouping

The primary outcome was an institutional composite pregnancy outcome as recorded in the unit’s electronic database. A positive outcome was defined as serum β-hCG positivity (≥25 mIU/mL measured 12–14 days after embryo transfer) with subsequent confirmation in the form of ongoing intrauterine pregnancy or live birth. A negative outcome comprised β-hCG negativity, biochemical pregnancy (transient β-hCG positivity without subsequent clinical confirmation), and ectopic pregnancy. This operational definition differs from the ESHRE/ICMART 2017 revised glossary in two respects: (i) sustained β-hCG positivity with subsequent clinical progression, rather than ultrasonographic visualization of a gestational sac per se, is used as the positive criterion; and (ii) ectopic pregnancies are classified within the negative category because they do not result in a viable intrauterine pregnancy. These definitional choices reflect the unit’s longitudinal electronic record coding rather than a prospective redefinition, and their limitations are acknowledged in Section 4.1.

Patients were stratified by number of oocytes retrieved into four groups: Group 1 (1–5 oocytes, *n* = 213), Group 2 (6–10, *n* = 376), Group 3 (11–15, *n* = 283), and Group 4 (≥16, *n* = 294). These groupings were defined a priori to reflect clinically interpretable response categories (poor, suboptimal, normal, high) broadly comparable to thresholds applied in earlier cohort analyses [7,9]; although alternative groupings (e.g., ≤ 3/4–9/10–15/≥ 16) have been used, the boundaries chosen here correspond to clinically meaningful response strata and yielded approximately balanced subgroup sizes. Infertility etiology was classified into five mutually exclusive categories according to the primary IVF indication recorded in the clinical charts: male factor, tubal factor, endometriosis, diminished ovarian reserve, and unexplained infertility.

### 2.5. Statistical Analysis

Continuous variables are expressed as mean ± standard deviation (or median with minimum–maximum for skewed variables), and categorical variables as counts and percentages. Normality was assessed with skewness/kurtosis and the Shapiro–Wilk test. Between-group comparisons used the independent-samples *t*-test or Mann–Whitney U test for continuous variables and the chi-square or Fisher exact test for categorical variables, as appropriate. Comparisons across the four oocyte groups used the Kruskal–Wallis test for continuous variables and chi-square for categorical variables, with the Cochran–Armitage test for ordered trend in pregnancy rates.

Discriminative performance of oocyte count for a positive pregnancy outcome was evaluated by receiver operating characteristic (ROC) analysis. The area under the curve (AUC) was calculated, and its 95% confidence interval was estimated using 2000-iteration non-parametric bootstrap resampling. The Youden index was used to identify the optimal cut-off. Univariate and multivariable logistic regression models were fitted with the composite pregnancy outcome as the dependent variable. The primary multivariable model included age, BMI, basal FSH, basal estradiol, AFC, infertility duration, stimulation duration, number of oocytes retrieved, number of embryos transferred, infertility etiology (dummy-coded with male factor as reference), and stimulation regimen (dummy-coded with rFSH monotherapy as reference). AMH was not included as AMH testing was gradually incorporated into routine practice during the study period and was therefore unavailable for a large portion of the cohort; AFC, which was available for nearly all participants and is strongly correlated with AMH, was retained as the primary ovarian reserve biomarker. Total gonadotropin dose was not included in the primary multivariable model as an a priori decision, because oocyte yield is a direct physiological consequence of gonadotropin exposure and embryologic competence; including both variables would have conflated the causal pathway from drug exposure to ovarian response, confounding the interpretation of oocyte count as a prognostic marker. A prespecified sensitivity analysis nonetheless refitted the model with both oocyte count and total gonadotropin dose as co-predictors. Model calibration was assessed with the Hosmer–Lemeshow goodness-of-fit test, and multicollinearity was evaluated using variance inflation factors (VIF).

To examine potential non-linearity in the association between oocyte count and the primary outcome, a restricted cubic spline with four knots placed at the 5th, 35th, 65th, and 95th percentiles was fitted, with adjustment for age and AFC. A likelihood ratio test compared the spline model with a linear-term model. Incremental discrimination of the full multivariable model over oocyte count alone was evaluated through three complementary approaches: (i) 2000-iteration bootstrap comparison of AUCs; (ii) integrated discrimination improvement (IDI); and (iii) continuous net reclassification improvement (NRI) per Pencina’s method [20]. Predefined subgroup analyses examined the oocyte-count–pregnancy relationship in patients below and at or above 35 years of age, in cycles using rFSH alone versus combined rFSH plus hMG, and across AFC tertiles (≤10, 11–18, >18). To characterize the precision of our null estimate, we assessed whether the 95% confidence interval of the adjusted odds ratio for oocyte count excluded a prespecified threshold of clinical meaningfulness (odds ratio ≥ 1.10 per additional oocyte), following a confidence-interval-based approach to establishing whether a clinically meaningful effect could be excluded in observational studies with null findings.

Missing data were limited (<1.5% for any included variable; detailed in Supplementary Table S3) and were handled by complete-case analysis. Given the low and non-differential missingness across the variables of interest, complete-case analysis was considered unlikely to introduce meaningful bias; no imputation was performed. The primary multivariable analysis was based on 1129 patients with complete records for all modeled variables; stratified oocyte-count analyses presented in Supplementary Table S2 were based on 1166 patients (excluding 5 patients with missing oocyte-count values). All bootstrap confidence intervals reported in this manuscript were derived from 2000 non-parametric resamples using the percentile method. Given the exploratory nature of subgroup and sensitivity analyses, formal multiple-testing correction was not applied; the consistently null pattern observed across the primary model, subgroup analyses, and spline modeling reinforces the robustness of the main finding. Two-sided *p* < 0.05 was considered statistically significant. Analyses were conducted using IBM SPSS Statistics version 25.0 (IBM Corp., Armonk, NY, USA) and Python 3.11 (Python Software Foundation) with the statsmodels, scipy, and scikit-learn libraries.

## 3. Results

### 3.1. Participant Characteristics

A total of 1171 patients met the inclusion criteria. A positive composite pregnancy outcome was observed in 430 patients (36.7%), while 741 (63.3%) had a negative outcome. Baseline demographic, hormonal, and cycle characteristics are presented in Table 1. Univariate crude odds ratios with 95% confidence intervals for each continuous predictor are presented alongside the descriptive statistics in Table 1 to facilitate interpretation; adjusted estimates from the multivariable model are presented in Section 3.3.

Patients who achieved a positive outcome were slightly younger than those who did not (30.74 ± 4.78 vs. 31.35 ± 5.46 years; *p* = 0.04). Antral follicle count was significantly higher in the positive-outcome group (16.02 ± 10.03 vs. 15.33 ± 9.57; *p* = 0.009). Total gonadotropin dose was slightly lower in the positive-outcome group than in the negative-outcome group (1978.23 ± 822.78 vs. 2085.03 ± 856.68 IU; *p* = 0.041), consistent with the common clinical practice of up-dosing in anticipated or observed suboptimal response; this univariate signal did not persist in multivariable analysis (sensitivity model; Section 3.3). Oocyte count showed a borderline univariate difference (11.87 ± 7.29 vs. 11.56 ± 6.58; *p* = 0.05). Groups did not differ in BMI, basal FSH, basal LH, basal estradiol, infertility duration, stimulation duration, or number of embryos transferred. Endometriosis was the most common infertility etiology (42.2% overall), with similar etiology distributions between outcome groups (*p* = 0.59). The distribution of stimulation regimens was comparable between groups (*p* = 0.36).

Two post-treatment cycle outcomes differed by pregnancy status: embryo cryopreservation was more frequent among patients achieving a positive outcome (85/430, 19.8%) than among those with a negative outcome (91/741, 12.3%; *p* = 0.001), consistent with better-quality embryologic cohorts in successful cycles. Hospitalization-requiring OHSS was infrequent in both groups and did not differ significantly (9/430, 2.1% vs. 8/741, 1.1%; *p* = 0.16). Because these variables reflect events downstream of the exposure of interest, they are reported here descriptively but were not entered as baseline covariates in subsequent modeling.

### 3.2. Cycle Outcomes by Oocyte Count

Characteristics of the four oocyte-count groups are shown in Table 2. Patient age decreased progressively across groups (from 34.40 ± 5.21 in Group 1 to 29.46 ± 4.55 years in Group 4; *p* < 0.001). Positive-outcome rates showed a modest numerical increase across groups (34.2%, 35.1%, 38.5%, and 39.5%, respectively), but the overall chi-square comparison was not significant (*p* = 0.59). The Cochran–Armitage test for trend yielded a borderline linear gradient of approximately 1.8 percentage points per group (*p* = 0.04), reflecting confounding by age and ovarian reserve rather than an independent oocyte effect, as confirmed by the multivariable analysis below. Embryo cryopreservation rates rose sharply across oocyte groups (4.2% in Group 1 to 24.8% in Group 4; *p* < 0.001), whereas the number of embryos transferred did not differ between groups (*p* = 0.44). Finer stratification of the ≥16-oocyte group is presented in Supplementary Table S2.

### 3.3. Univariate and Multivariable Logistic Regression

In univariate logistic regression, higher basal FSH was associated with a lower probability of a positive outcome (crude odds ratio [cOR] 0.95, 95% confidence interval [CI] 0.91–0.99; *p* = 0.03), whereas higher AFC was associated with a higher probability (cOR 1.02, 95% CI 1.00–1.03; *p* = 0.03). Age showed a trend toward inverse association (cOR 0.98, *p* = 0.05). Oocyte count showed no significant crude association (cOR 1.02, 95% CI 1.00–1.03; *p* = 0.08).

After multivariable adjustment (Table 3), the number of oocytes retrieved was not independently associated with the primary outcome in the adjusted model used here (adjusted odds ratio [aOR] 0.999, 95% CI 0.979–1.020; *p* = 0.96). None of the covariates reached formal statistical significance, although age demonstrated a borderline inverse association (aOR 0.97, 95% CI 0.94–1.00; *p* = 0.06). The model demonstrated adequate calibration (Hosmer–Lemeshow χ^2^ = 5.30, *p* = 0.73), no evidence of multicollinearity (all VIF < 1.6 in the primary model), and an events-per-variable ratio of 26.9, well above the recommended threshold of 10–20 [21]. In a prespecified sensitivity analysis including both oocyte count and total gonadotropin dose as co-predictors (oocyte–dose Pearson r = −0.30; VIF = 1.45 and 2.06, respectively), neither variable reached statistical significance (oocyte count aOR 0.999, 95% CI 0.979–1.020, *p* = 0.93; total dose aOR 1.000, 95% CI 1.000–1.000, *p* = 0.08), confirming the robustness of the primary finding and indicating that the univariate association of total dose with outcome (Section 3.1) is confounded by age and ovarian reserve. In an extended dose-stratified subgroup analysis prepared in response to peer review (Supplementary Table S5), patients were further stratified into three pre-specified total-dose categories (<1500 IU, 1500–3000 IU, and ≥3000 IU). Within each dose-category subgroup, a reduced multivariable model adjusting for age and antral follicle count yielded an oocyte-count adjusted odds ratio close to unity and statistically non-significant (low dose: aOR 1.006, 95% CI 0.974–1.038, *p* = 0.73; mid dose: aOR 1.005, 95% CI 0.980–1.031, *p* = 0.69; high dose: aOR 0.989, 95% CI 0.920–1.063, *p* = 0.76), with a non-significant 3-group chi-square test for positive-outcome rates (*p* = 0.11) and a borderline ordered-category trend (Cochran–Armitage *p* = 0.061) consistent with confounding by clinical indication. Starting-dose categorization was not feasible because the starting dose was not captured as a separate variable in the institutional database.

### 3.4. Discriminative Performance and Reclassification Analyses

**Figure 1:**
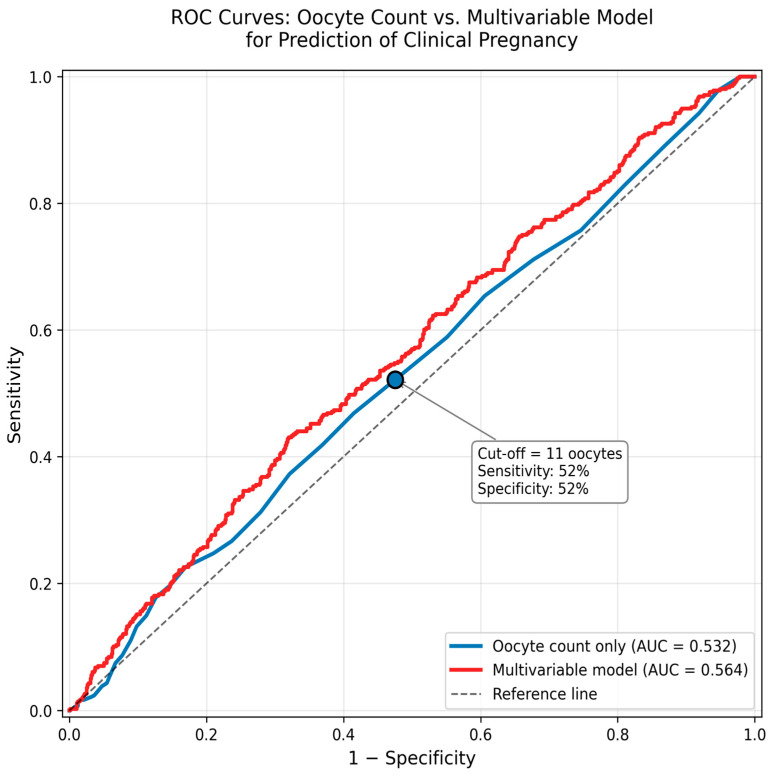
Receiver operating characteristic (ROC) curves for prediction of a positive primary outcome by oocyte count alone (blue) and by the full multivariable logistic regression model (red). The Youden-optimal cut-off for oocyte count was 11 oocytes retrieved, yielding sensitivity of 52% and specificity of 52%. The area under the curve (AUC) was 0.532 (bootstrap 95% confidence interval 0.498–0.567) for oocyte count alone and 0.564 (bootstrap 95% confidence interval 0.529–0.598) for the multivariable model; the between-model AUC difference was 0.031 (bootstrap *p* = 0.11). Complementary reclassification analyses (integrated discrimination improvement 0.011, continuous net reclassification improvement 0.135) confirmed a statistically detectable but clinically trivial incremental discrimination (Supplementary Table S1).

Oocyte count alone yielded a ROC AUC of 0.532 (bootstrap 95% CI 0.498–0.567), indicating performance only marginally superior to random classification (Figure 1). At the Youden-optimal cut-off of 11 oocytes retrieved, sensitivity and specificity were 52% and 52%, respectively. The full multivariable model achieved an AUC of 0.564 (bootstrap 95% CI 0.529–0.598). The AUC increment of 0.031 in favor of the multivariable model did not reach statistical significance (bootstrap *p* = 0.11). Complementary reclassification analyses confirmed that any incremental discrimination beyond oocyte count alone was statistically detectable but clinically trivial: IDI was 0.011 (95% CI 0.005–0.017; bootstrap *p* < 0.001), indicating an absolute probability difference of roughly one percentage point between predicted probabilities in events and non-events; continuous NRI was 0.135 (95% CI 0.014–0.251; bootstrap *p* = 0.03), a small-to-moderate improvement consistent with marginal reclassification. From a clinical utility standpoint, a multivariable model with AUC of 0.56 offers negligible decisional advantage over chance, reinforcing the conclusion that neither oocyte count alone nor its inclusion in a broader predictor set achieves a level of discriminative performance useful for clinical decision-making (Supplementary Table S1).

### 3.5. Non-Linear Dose–Response Analysis

**Figure 2:**
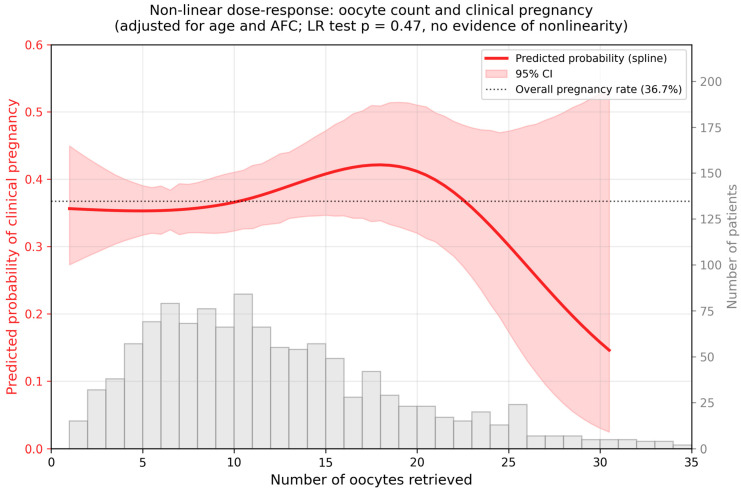
Restricted cubic spline regression of the association between number of oocytes retrieved and predicted probability of a positive primary outcome, adjusted for age and antral follicle count (red line: predicted probability; shaded band: 95% confidence interval; dotted line: overall positive-outcome rate of 36.7%). The light-gray histogram shows the distribution of oocytes retrieved in the cohort. Although the spline trajectory visually suggests a concave pattern with a nominal peak predicted probability around 17–18 oocytes, the likelihood ratio test comparing the spline model with a linear-term model was non-significant (χ^2^ = 2.52, df = 3; *p* = 0.47), indicating no statistical evidence of a non-linear threshold or plateau across the observed range; the visual pattern should be regarded as hypothesis-generating rather than as a confirmed dose–response feature. The widening confidence band at the right tail reflects the smaller number of patients at very high oocyte yields.

Restricted cubic spline regression of oocyte count against the primary outcome, adjusted for age and AFC, revealed no statistically significant non-linearity (likelihood ratio test: χ^2^ = 2.52, df = 3; *p* = 0.47). Predicted probabilities remained within 35–42% across the bulk of the observed range (5–22 oocytes, where data density is highest), with wide confidence bands at the upper extremes reflecting smaller sample sizes (Figure 2). The adjusted spline trajectory visually suggested a modest concave pattern with a nominal peak predicted probability around 17–18 oocytes; however, because the likelihood-ratio test was non-significant, this visual pattern should be interpreted as hypothesis-generating rather than confirmatory and not as empirical evidence of a threshold effect.

### 3.6. Subgroup Analyses

In age-stratified analyses, the AUC of oocyte count for a positive outcome was 0.52 (95% CI 0.48–0.55) in patients aged <35 years (*n* = 847) and 0.55 (95% CI 0.48–0.61) in patients aged ≥35 years (*n* = 319). In regimen-stratified analyses, the AUC was 0.53 in cycles using rFSH alone (*n* = 625) and 0.55 in cycles using rFSH plus hMG (*n* = 476). In an extended regimen-stratified analysis prepared in response to peer review (Supplementary Table S4), a reduced multivariable model adjusting for age and antral follicle count was additionally fitted within each of the four regimen subgroups; the adjusted odds ratio for oocyte count remained close to unity and statistically non-significant within the rFSH-alone (aOR 1.007, 95% CI 0.984–1.032, *p* = 0.55), rFSH + hMG (aOR 1.011, 95% CI 0.976–1.047, *p* = 0.56), and hMG (aOR 0.933, 95% CI 0.793–1.097, *p* = 0.40) subgroups, with the small rFSH + recLH subgroup (*n* = 6) precluding a stable model fit; the unadjusted four-group chi-square comparison of positive-outcome rates was also non-significant (*p* = 0.36, Table 1), supporting the regimen-independence of the principal null finding.

In AFC-stratified analyses using tertile cut-offs (AFC ≤ 10, 11–18, >18), the AUC of oocyte count was 0.534 (95% CI 0.476–0.589) in the lowest AFC tertile (*n* = 435), 0.527 (95% CI 0.470–0.589) in the middle tertile (*n* = 352), and 0.472 (95% CI 0.415–0.534) in the highest AFC tertile (*n* = 370). All three confidence intervals crossed 0.50, indicating no statistically significant subgroup-specific discrimination. The numerically inverse direction of the AUC in the highest AFC tertile (i.e., higher oocyte counts associated with lower predicted probability of a positive outcome) is descriptively consistent with a ceiling-type pattern whereby, beyond an adequate follicular reserve, additional retrieved oocytes may not contribute to—and, descriptively, may inversely reflect—fresh-cycle pregnancy probability; however, because the confidence interval crosses 0.50, this observation is hypothesis-generating rather than confirmatory and should not be read as evidence for any particular mechanistic explanation. Across age and regimen subgroups, the AUC remained close to 0.50, indicating weak discriminative performance; in the highest AFC tertile, the numerically inverse AUC (0.472) is qualitatively distinct and should be interpreted as a ceiling-consistent descriptive signal rather than as generalized null discrimination. Subgroup ROC analyses were restricted to patients with complete oocyte-count and subgroup-defining-variable data; subgroup denominators therefore differ slightly from the full cohort of 1171.

### 3.7. Precision of the Null Estimate

To characterize the precision of our null estimate, we examined the 95% confidence interval of the adjusted odds ratio for oocyte count. The interval (0.979–1.020) excluded, at its upper bound, any per-oocyte effect equal to or greater than the conventional threshold of clinical meaningfulness (odds ratio ≥ 1.10). With *n* = 1129 in the multivariable analysis and an event rate of 36.8%, our analysis had sufficient precision to exclude a clinically meaningful independent per-oocyte effect within the limits of the adjusted model, supporting the absence of a materially important independent association within this dataset rather than an inconclusive finding arising from inadequate statistical power. This inference is conditional on the predictors and outcome definition used here; effects mediated by variables not captured in our dataset (embryo morphology, transfer day, live birth) cannot be addressed by the present analysis.

## 4. Discussion

In this single-center retrospective cohort of 1171 women undergoing their first GnRH antagonist IVF cycle with fresh embryo transfer, the number of oocytes retrieved demonstrated weak discriminative performance for a positive composite pregnancy outcome (AUC 0.53) and, after multivariable adjustment for age, ovarian reserve, stimulation characteristics, infertility etiology, and regimen, was not independently associated with the primary outcome in the adjusted model used here (aOR 0.999, *p* = 0.96). Complementary reclassification analyses (IDI 0.011; continuous NRI 0.135) indicated that any incremental discrimination achieved by the multivariable model over oocyte count alone was statistically detectable but of trivial magnitude. Restricted cubic spline regression revealed no statistically significant non-linear threshold or plateau, and findings were consistent across age, stimulation regimen, and AFC subgroups. The 95% confidence interval of the adjusted odds ratio (0.979–1.020) excluded any per-oocyte effect of odds ratio ≥1.10, indicating that our sample had sufficient precision to exclude a clinically meaningful independent effect. These inferences, however, are conditional on the predictors and outcome captured in our dataset: we did not have access to embryo morphology, transfer day, or live-birth outcomes, and our primary endpoint is an institutional composite pregnancy outcome (Section 2.4) rather than the ESHRE/ICMART-standard clinical pregnancy or live birth. Our findings should therefore be read as suggesting that oocyte yield alone is a weak standalone prognostic marker of fresh-cycle success within the analytic framework used here, rather than as evidence that oocyte count is unimportant for IVF outcome in general.

The unadjusted comparisons reported above and the cut-off analysis (Section 3.4) suggested a numerically higher pregnancy rate above an oocyte threshold of 11. Multivariable adjustment, however, indicates that this pattern is largely explained by age and ovarian reserve, which distribute asymmetrically across oocyte-yield strata (mean age 34.4 years in Group 1 vs. 29.4 years in Group 4); once these covariates are jointly modeled, the independent contribution of oocyte count is no longer detected (aOR 0.999, *p* = 0.96). This trajectory illustrates why unadjusted threshold findings in IVF cohort data warrant cautious interpretation alongside multivariable adjustment.

Our results contribute data that are specific to the GnRH antagonist protocol, in which evidence regarding the optimal oocyte yield has historically been more limited than for long agonist cycles. The large pooled analyses of Sunkara et al. [7], Ji et al. [8], and Van der Gaast et al. [9], while methodologically influential, included predominantly mixed or long agonist cohorts and reported optimum yields in the range of 13–15 oocytes for live birth. Notably, Datta et al. [22] reported that in mild stimulation IVF cycles, live birth rate per cycle plateaued at approximately nine oocytes while cumulative live birth rate optimized at twelve—findings broadly consistent with the pattern we observed in our GnRH antagonist cohort. Similarly, in a large Swedish registry analysis of 77,956 IVF cycles, Magnusson et al. [23] observed that live birth rate per fresh cycle plateaued and began to decline beyond approximately 11 oocytes, while the incidence of ovarian hyperstimulation syndrome rose sharply above 18 oocytes—highlighting that the pursuit of ever-higher oocyte yields may yield diminishing returns in pregnancy outcomes while escalating safety risks. Mechanistic support for the view that oocyte quantity alone is not the limiting determinant comes from donor-oocyte studies, in which gamete quality is relatively standardized; Letterie et al. [24] reported that within donor cycles, the strength of the association between oocyte number and successful outcome was modest, consistent with the interpretation that factors beyond oocyte yield—including embryo competence and endometrial receptivity—substantially mediate the pregnancy outcome. More recent antagonist-specific literature has painted a more nuanced picture. In a prospective study specifically within GnRH antagonist cycles, Tsakos et al. [25] evaluated the predictive performance of AMH, FSH, and AFC for ovarian response and reported that pre-treatment ovarian reserve markers stratified patients into poor, normal, and high responder categories more consistently than oocyte yield alone, supporting the interpretation that baseline ovarian reserve rather than the final oocyte count carries the principal prognostic information within antagonist cycles. Consistent with this, Zhao et al. [26] and Xu et al. [27] addressed different modifications to the GnRH antagonist protocol—reduced antagonist dosing and antagonist early cessation with gonadotropin step-down, respectively—yet both studies reinforce the broader message that in antagonist cycles, pregnancy outcomes are driven more by overall stimulation optimization and individual patient factors than by oocyte yield per se; our findings extend this message by demonstrating the same pattern using a direct analytic focus on oocyte count as a prognostic marker. In line with this interpretation, our regimen-stratified subgroup analysis (Supplementary Table S4) demonstrated that the null adjusted association between oocyte count and pregnancy was robust across the principal stimulation-regimen subgroups represented in the cohort, indicating that the choice of gonadotropin preparation (rFSH alone, rFSH + hMG, or hMG) did not by itself confound the principal finding. Within the POSEIDON framework, the central premise that patient stratification (age, ovarian reserve, prior response) is more informative than oocyte yield alone [13,14] is consistent with our adjusted null and with large contemporary cohorts that document fresh-cycle pregnancy plateau or decline at higher yields even while cumulative live-birth rates continue to accrue [15]. Within this individualization paradigm, adjunctive strategies such as recombinant LH supplementation in patients with hypo-response to standard stimulation have been proposed to improve outcomes in low-prognosis subgroups [28], complementing the broader argument that quantitative oocyte yield should be interpreted in the context of individualized ovarian reserve and prior response rather than treated as a standalone predictor.

The borderline linear trend in unadjusted pregnancy rates across quartile-like oocyte groups (1.8 percentage points per group; *p* = 0.04) is consistent with prior crude comparisons. Importantly, however, this trend disappeared after adjustment (aOR 0.999; *p* = 0.96), indicating that the apparent group-level association is driven by confounding—principally by age, with progressively younger women populating the higher-yield groups (34.4 years in Group 1 vs. 29.4 years in Group 4). The quantitative dissociation between unadjusted and adjusted estimates illustrates why multivariable modeling is essential when interpreting observational data on oocyte yield and pregnancy outcomes.

Both age and AFC showed the expected univariate associations with the primary outcome but attenuated in the multivariable model (Table 3). This pattern does not reflect model over-parameterization: variance inflation factors for all predictors remained below 1.6, and the events-per-variable ratio (26.9) was well above the recommended threshold of 10–20 [21]. Rather, the attenuation reflects the well-known biological interdependence of age, ovarian reserve, and oocyte yield, which share variance because they reflect overlapping dimensions of ovarian aging. When modeled jointly, their marginal contributions are partitioned, and the strongest individual signals from simple univariate comparisons are redistributed across correlated covariates. This is a reproducible phenomenon in the IVF prediction literature [29,30] and does not indicate a statistical artifact; it does, however, underscore that age and ovarian reserve should be interpreted as a unified prognostic dimension rather than as truly independent predictors.

One biologically motivated framework for interpreting the absence of a simple monotonic dose–response relationship is that markedly elevated estradiol levels during high-response stimulation may adversely affect endometrial receptivity and embryo–endometrial synchrony, potentially counterbalancing quantitative gains from additional oocytes [10,11]; in addition, milder stimulation has been associated with reduced embryonic aneuploidy in a randomized setting [12]. Our data are not a formal test of these mechanisms. Three descriptive observations are nonetheless compatible with, but do not confirm, a ceiling-type interpretation: a visually concave (but statistically non-significant) spline trajectory with a nominal peak around 17–18 oocytes; a numerically inverse direction of oocyte-count discrimination in the highest AFC tertile (AUC 0.47, 95% CI 0.42–0.53, crossing 0.50); and, in finer stratification (Supplementary Table S2), a descriptive peak in the 21–25 oocyte stratum (unadjusted, displaced from the adjusted spline peak because high-oocyte strata contain disproportionately younger patients). None of these observations reaches conventional statistical significance (spline LR *p* = 0.47; high-AFC AUC CI crosses 0.50; six-group χ^2^ *p* = 0.059), and we therefore present them strictly as hypothesis-generating. Confirmatory evaluation would require prospective designs with standardized outcomes and embryo-level covariates.

A notable observation is that embryo cryopreservation was significantly more frequent in higher-yield groups (from 4.2% in Group 1 to 24.8% in Group 4; *p* < 0.001) and in patients who achieved a positive outcome (19.8% vs. 12.3%; *p* = 0.001). This reflects the well-documented contribution of high ovarian response to the cumulative live-birth potential achievable after a single stimulation [15,31,32]. Thus, while our findings do not support oocyte yield as a predictor of fresh-cycle pregnancy, a higher yield remains clinically valuable in extending cumulative success through supernumerary cryopreserved embryos. At the same time, the pursuit of maximal oocyte yield must be balanced against the historically well-documented risks of aggressive stimulation—including multiple pregnancy, which carries substantial maternal and neonatal morbidity and has long been recognized as a core safety concern of ovarian stimulation [33]—reinforcing the rationale for single embryo transfer and judicious stimulation intensity in contemporary practice.

A further methodological consideration concerns the exclusion of freeze-all cycles. This was not an inadvertent selection but a deliberate scoping decision: our research question specifically addresses the predictive value of oocyte count for fresh-cycle clinical pregnancy, which remains a clinically relevant outcome in contemporary practice. Importantly, high-yield patients who proceeded to fresh transfer were retained in the cohort (294 patients with ≥16 oocytes, with pregnancy rates of 34.9%, 51.7%, and 32.2% in the 16–20, 21–25, and ≥26 strata, respectively; Supplementary Table S2), and the null adjusted association persisted within these high-response strata. Our findings therefore apply to the substantial clinical practice in which fresh-cycle transfer is chosen, and should not be extrapolated to the cumulative outcomes of integrated fresh-plus-frozen strategies. Cumulative live-birth analyses incorporating frozen-embryo transfers represent an important and complementary research question that should be addressed in future multicenter prospective studies.

An important covariate not captured in our dataset is embryo morphological quality at transfer (e.g., Gardner grading for blastocysts, or day-3 cleavage-stage grading). The absence of granular embryo-quality data is a clear limitation: if embryo quality is the dominant determinant of fresh-cycle pregnancy—as suggested by contemporary single-embryo transfer literature—then the modest association we observed between oocyte count and pregnancy, and its attenuation after multivariable adjustment, may be further mediated by embryo-level heterogeneity. Future studies should incorporate embryo morphology and, where available, time-lapse morphokinetic parameters to disentangle oocyte-level quantity from embryo-level quality contributions.

### 4.1. Strengths and Limitations

Strengths of this study include a large single-center cohort (*n* = 1171) representing the complete historical population of the unit from the inception of its assisted reproduction services through the study design date, restriction to first IVF cycles with a single class of stimulation (GnRH antagonist) to minimize between-protocol heterogeneity, complete ascertainment of cycle and outcome variables from a unified institutional database, a wide age range enhancing generalizability, and a layered analytic approach comprising multivariable logistic regression, bootstrap-based ROC comparison, IDI and NRI, restricted cubic spline modeling, predefined subgroup analyses (including AFC tertiles), sensitivity analysis with total gonadotropin dose, and a confidence-interval-based precision analysis of the null estimate. These methods collectively provide a broader analytic characterization of the predictive performance of oocyte count than is routinely available in prior single-center reports on this question.

Several limitations should be acknowledged. First, the retrospective single-center design limits causal inference and may limit generalizability. Second, the primary outcome was an institutional composite pregnancy outcome that differs from the ESHRE/ICMART 2017 revised glossary; although this represents the outcome recorded in our longitudinal database, standardized outcomes such as live birth would be more definitive endpoints. Population-level live-birth ascertainment was not systematically linked to the ART unit electronic database during the study period and therefore could not be reconstructed retrospectively for the full cohort; pregnancy outcome as defined above was the most granular endpoint reliably available. Specifically, a sensitivity analysis restricted to cases with documented ultrasonographic gestational-sac visualization—which would have allowed a more conservative effect estimate at an earlier endpoint along the pregnancy trajectory—was not feasible retrospectively, because the institutional database recorded the primary outcome as a single composite binary variable without preserving the sub-classification between ultrasonographic clinical pregnancy and ongoing pregnancy/live birth at the case level. This sensitivity analysis is also precluded prospectively by the closure of the original treating institution in 2025 (described in the following paragraph). Future prospective studies with separately recorded sonographic and ongoing-pregnancy endpoints will be required to refine effect estimates at the ultrasound-confirmed clinical-pregnancy level. Third, AMH was not uniformly available throughout the study period and was therefore not included in multivariable models; however, AFC was available for nearly the entire cohort and served as the primary ovarian reserve biomarker. Fourth, embryo transfer day (day 2, 3, or 5) was not separately recorded in the institutional database during the study period and therefore could not be incorporated as a covariate in the multivariable analysis, stratified across in subgroup comparisons, or used to perform a between-group (cleavage-stage vs. blastocyst-stage transfer) outcome comparison. Although blastocyst-stage transfers have been associated with higher implantation potential per transferred embryo than cleavage-stage transfers in randomized and observational studies, accumulated evidence from a Cochrane systematic review suggests that the differential between the two transfer strategies, in terms of cumulative pregnancy outcomes from a single oocyte retrieval, is more modest than per-transfer-cycle comparisons alone would suggest [34]. This database constraint cannot be remedied retrospectively by additional source-level extraction owing to the closure of the original treating institution in 2025 (described in the following paragraph), and represents residual heterogeneity in the present analysis; however, given that the stimulation backbone, oocyte retrieval protocol, and universal ICSI application were invariant throughout the study period, transfer-day heterogeneity is unlikely to selectively distort the principal null association between oocyte count and the composite pregnancy outcome. Relatedly, the specific trigger preparation administered (recombinant versus urinary hCG) was not retained as a separate categorical variable in the longitudinal database and therefore could not be incorporated as a covariate in the multivariable model or stratified across in subgroup analyses; this constraint is mitigated by evidence from a Cochrane systematic review of randomized trials reporting comparable ongoing-pregnancy and live-birth outcomes for the two preparations [19], suggesting that residual confounding from trigger-preparation heterogeneity is unlikely to be substantial. Fifth, universal ICSI application regardless of infertility etiology reflects institutional practice during the study period; although this conflicts with current ASRM/ESHRE recommendations restricting ICSI to male-factor or prior-fertilization-failure indications, it confers methodological homogeneity by eliminating fertilization-method heterogeneity as a confounder. The generalizability of our findings to units using conventional IVF for non-male-factor cases should therefore be considered cautiously.

A structural limitation that affects several of the analytical choices above concerns data accessibility. Ankara Etlik Zübeyde Hanım Women’s Health Training and Research Hospital, the institution in which the present cohort was accrued, was permanently closed in 2025. Consequently, the original longitudinal ART database is no longer accessible, and retrospective re-extraction of additional variables—in particular population-level live-birth ascertainment, precise embryo-transfer day, embryo morphology grading, and calendar-year-stratified analyses—is no longer feasible. The present dataset represents the most complete institutional record retrievable prior to closure, and our findings should be interpreted within these structural constraints.

A further concern in any 14-year cohort is secular change: during this period, cryopreservation shifted from slow-freezing toward vitrification, blastocyst culture became more common, trigger choices expanded, and incubator technologies and culture-media formulations evolved substantially. Two features of our study design attenuate this concern. First, the stimulation backbone (GnRH antagonist with rFSH-based ovarian stimulation) and the oocyte retrieval protocol (transvaginal aspiration 34–36 h after trigger, ICSI in all cases) were invariant throughout the study period. Second, our predefined subgroup analyses across age strata, stimulation regimens, and AFC tertiles yielded uniformly weak discrimination of oocyte count (AUC range 0.47–0.55), indicating that the null association is robust to clinical heterogeneity that plausibly parallels temporal heterogeneity. The net direction of any residual era-related bias, however, cannot be empirically determined without calendar-year stratification, which was not feasible following institutional database closure as described above. Potential era-related influences—including expanded cryopreservation, blastocyst culture, refinements in incubation and culture-media technology, and embryo-selection tools—could bias the observed association in either direction. A further consideration is the tertiary referral nature of the source institution, which may have concentrated patients with more complex or refractory infertility phenotypes relative to community IVF practice; this setting-specific case mix should be borne in mind when generalizing our findings. Our results should therefore be interpreted as applicable to contemporary antagonist IVF practice at a tertiary referral center broadly, rather than to any specific technological era or patient population.

### 4.2. Clinical Implications

Our findings have several tentative implications that should be interpreted in the context of the study’s limitations. First, counseling based on oocyte yield alone may provide limited prognostic information for fresh-cycle pregnancy in antagonist IVF, particularly when established parameters such as age and AFC are also considered; nonetheless, the Youden-optimal cut-off of 11 oocytes identified in our data is consistent with the clinical practice of targeting an adequate oocyte yield as a descriptive benchmark of ovarian response. Second, although the univariate signal for total gonadotropin dose did not persist in multivariable analysis, stimulation strategies that prioritize maximization of oocyte number as a primary goal may not correspond to proportional fresh-cycle pregnancy gains and should be weighed against known risks such as OHSS. Third, because embryo cryopreservation rates rise substantially with oocyte yield, a higher oocyte response may contribute to cumulative rather than fresh-cycle outcomes—a distinction that warrants explicit attention in counseling. These implications are hypothesis-generating rather than practice-changing; prospective multicenter studies using live birth as the primary endpoint, incorporating AMH and embryo morphology, and employing modern predictive-modeling approaches are needed before oocyte-count-based thresholds are revised in clinical guidance.

## 5. Conclusions

In this large single-center retrospective cohort of GnRH antagonist IVF cycles, the number of oocytes retrieved was not independently associated with a positive fresh-cycle pregnancy outcome in the adjusted model used here, and its discriminative performance was weak both in isolation and when combined with other clinical predictors. The precision of our estimates was sufficient to exclude a large per-oocyte effect within the limits of the adjusted model; however, we cannot exclude smaller effects or effects that would emerge if embryo morphology, transfer day, and live-birth outcomes were available. Our findings should therefore not be read as evidence that oocyte count is unimportant in IVF; rather, they suggest that within antagonist IVF cycles, oocyte yield alone is a weak standalone prognostic marker of fresh-cycle success and should be interpreted alongside—rather than as a substitute for—established clinical parameters such as age, ovarian reserve, and, in future analyses, embryo quality. The principal clinical value of a higher oocyte response in this setting may lie in cumulative rather than fresh-cycle success, a hypothesis that warrants prospective investigation using standardized outcomes.

Nevertheless, the unadjusted gradient across oocyte-yield strata (Cochran-Armitage trend *p* = 0.04) and the Youden-optimal cut-off of 11 oocytes are consistent with the clinical practice of targeting an adequate ovarian response, and the marked rise in embryo cryopreservation at higher yields (4.2% to 24.8%, *p* < 0.001) supports the contribution of a higher response to cumulative rather than fresh-cycle success.

## Figures and Tables

**Table 1:** Baseline demographic, hormonal, and cycle characteristics by primary outcome status.

	Positive Outcome (*n* = 430, 36.7%)	Negative Outcome (*n* = 741, 63.3%)	Univariate cOR (95% CI)	*p*-Value
Mean ± SD	Mean ± SD	Mean ± SD		
Age (years)	30.74 ± 4.78	31.35 ± 5.46	0.978 (0.956–1.000)	0.04 *
Infertility duration (months)	74.60 ± 50.58	69.54 ± 48.09	1.002 (1.000–1.005)	0.09 *
BMI (kg/m^2^)	26.74 ± 4.93	26.85 ± 4.92	0.995 (0.971–1.020)	0.69 *
Basal FSH (IU/L)	7.73 ± 2.69	8.03 ± 4.19	0.952 (0.911–0.995)	0.61 ^†^
Basal LH (IU/L)	5.47 ± 3.09	5.72 ± 2.99	1.005 (0.969–1.043)	0.79 ^†^
Basal E2 (ng/L)	46.84 ± 18.35	45.18 ± 18.06	1.002 (0.996–1.008)	0.35 ^†^
AFC	16.02 ± 10.03	15.33 ± 9.57	1.015 (1.002–1.028)	0.009 ^†^
Stimulation duration (days)	10.13 ± 1.55	10.02 ± 1.50	1.056 (0.981–1.137)	0.42 ^†^
Total gonadotropin dose (IU)	1978.23 ± 822.78	2085.03 ± 856.68	1.000 (1.000–1.000)	0.041 ^†^
Oocytes retrieved	11.87 ± 7.29	11.56 ± 6.58	1.015 (0.998–1.031)	0.05 ^†^
Embryos transferred	1.25 ± 0.43	1.24 ± 0.43	1.170 (0.948–1.446)	0.10 ^†^
*n* (%)	*n* (%)	*n* (%)		
Infertility cause				0.59 ^‡^
Endometriosis	194 (45.1%)	300 (40.5%)	—	
Male factor	103 (24.0%)	182 (24.6%)	—	
Diminished ovarian reserve	85 (19.8%)	168 (22.7%)	—	
Tubal factor	33 (7.7%)	62 (8.4%)	—	
Unexplained	15 (3.5%)	29 (3.9%)	—	
Stimulation regimen				0.36 ^‡^
rFSH	243 (56.5%)	383 (51.7%)	—	
rFSH + hMG	163 (37.9%)	315 (42.5%)	—	
hMG	21 (4.9%)	40 (5.4%)	—	
rFSH + recLH	3 (0.7%)	3 (0.4%)	—	

Values are mean ± standard deviation unless otherwise indicated. Positive outcome: sustained β-hCG positivity with subsequent ongoing intrauterine pregnancy or live birth. Negative outcome: β-hCG negativity, biochemical pregnancy, or ectopic pregnancy. Univariate cOR: crude (unadjusted) odds ratio per natural unit of the predictor (per year, per kg/m^2^, per IU/L, etc.), with 95% confidence interval, from per-variable complete-case univariate logistic regression. For categorical variables (infertility cause, stimulation regimen), univariate logistic regression was not performed in this table; their independent contributions, dummy-coded with male factor and rFSH monotherapy as references, are reported in the multivariable model (Section 3.3). * Independent-samples *t*-test. † Mann–Whitney U test. ‡ Pearson chi-square test. AFC: antral follicle count; BMI: body mass index; cOR: crude odds ratio; CI: confidence interval; E2: estradiol; FSH: follicle-stimulating hormone; hMG: human menopausal gonadotropin; LH: luteinizing hormone; rFSH: recombinant follicle-stimulating hormone; recLH: recombinant luteinizing hormone. Post-treatment variables (embryo cryopreservation and hospitalization-requiring OHSS) are reported in the Results text (Section 3.1) rather than in this baseline table.

**Table 2:** Cycle characteristics and primary outcome rates according to oocyte-count group.

	Group 1 (1–5) *n* = 213	Group 2 (6–10) *n* = 376	Group 3 (11–15) *n* = 283	Group 4 (≥16) *n* = 294	*p*
Age (years)	34.40 ± 5.21	31.32 ± 5.25	30.07 ± 4.68	29.46 ± 4.55	<0.001 *
Oocytes retrieved	3.62 ± 1.28	8.03 ± 1.45	12.89 ± 1.42	22.06 ± 5.51	<0.001 *
Embryos transferred	1 (1–3)	1 (1–3)	1 (1–3)	1 (1–3)	0.44 *
Cryopreservation, *n* (%)	9 (4.2%)	43 (11.4%)	51 (18.0%)	73 (24.8%)	<0.001 ^†^
Positive outcome, *n* (%)	73 (34.2%)	132 (35.1%)	109 (38.5%)	116 (39.5%)	0.59 ^†^

Continuous variables are expressed as mean ± standard deviation, or median (minimum–maximum) for skewed variables. * Kruskal–Wallis test. † Pearson chi-square test. Percentages are calculated within each oocyte-count group. Five patients with missing oocyte-count values were excluded from this stratified analysis (*n* = 1166 of 1171); see Supplementary Table S2 for finer stratification of the ≥16-oocyte group (16–20, 21–25, ≥26).

**Table 3:** Multivariable logistic regression for predictors of a positive primary outcome (*n* = 1129).

Predictor	aOR	95% CI	*p*-Value
Age (years)	0.972	0.944–1.001	0.059
BMI (kg/m^2^)	0.992	0.967–1.018	0.546
Basal FSH (IU/L)	0.973	0.932–1.017	0.229
Basal E2 (ng/L)	1.003	0.997–1.009	0.338
AFC	1.007	0.989–1.025	0.458
Infertility duration (months)	1.002	0.999–1.004	0.236
Stimulation duration (days)	1.039	0.960–1.125	0.345
Oocytes retrieved	0.999	0.979–1.020	0.961
Embryos transferred	1.211	0.950–1.544	0.123
Infertility cause (ref: Male factor)			
Tubal factor	0.914	0.551–1.516	0.727
Endometriosis	1.047	0.767–1.430	0.772
Unexplained	1.271	0.595–2.716	0.536
Diminished ovarian reserve	1.083	0.722–1.623	0.701
Stimulation regimen (ref: rFSH)			
rFSH + hMG	0.994	0.735–1.344	0.969
hMG	1.171	0.624–2.195	0.624
rFSH + recLH	1.375	0.267–7.092	0.704

Model calibration: Hosmer–Lemeshow χ^2^ = 5.30, *p* = 0.73 (adequate fit). Discriminative performance: AUC = 0.564 (bootstrap 95% CI 0.529–0.598, percentile method, 2000 iterations). Multicollinearity: all variance inflation factors <1.6 in the primary model; events-per-variable ratio = 26.9, exceeding the recommended threshold of 10–20 [21]. AMH was not included as a covariate because AMH testing was not routinely available throughout the 14-year study period; AFC was used as the primary ovarian reserve biomarker. Total gonadotropin dose was not included in the primary model to avoid conflating the exposure–response pathway from drug administration to oocyte yield; a prespecified sensitivity analysis including total dose yielded consistent results (oocyte count aOR 0.999, *p* = 0.93; total dose aOR 1.000, *p* = 0.08; oocyte–dose Pearson *r* = −0.30; VIF 1.45 and 2.06, respectively). Units of measurement: continuous predictors are modeled per natural unit (age per year, BMI per kg/m^2^, basal FSH per IU/L, basal E2 per ng/L, AFC per follicle, infertility duration per month, stimulation duration per day, oocytes retrieved per oocyte, embryos transferred per embryo). aOR: adjusted odds ratio; AFC: antral follicle count; BMI: body mass index; CI: confidence interval; E2: estradiol; FSH: follicle-stimulating hormone; hMG: human menopausal gonadotropin; rFSH: recombinant follicle-stimulating hormone; recLH: recombinant luteinizing hormone.

## Data Availability

The data that support the findings of this study are not publicly available owing to institutional confidentiality constraints and the permanent closure of the source institution in 2025, but de-identified aggregate data may be made available from the corresponding author upon reasonable request and with institutional approval.

## Supplementary Materials

The following supporting information can be downloaded at: https://www.mdpi.com/article/10.3390/medicina62061110/s1, Supplementary Table S1: Discriminative performance and reclassification analyses comparing oocyte count alone versus the full multivariable logistic regression model; Supplementary Table S2: Fine-grained stratification of oocyte counts and associated positive-outcome rates in the high-response cohort; Supplementary Table S3: Missing data summary for variables used in the primary multivariable analysis; Supplementary Table S4: Stimulation-regimen subgroup analysis showing descriptive outcomes and oocyte-count adjusted odds ratios within each regimen subgroup; Supplementary Table S5: Total gonadotropin dose subgroup analysis showing descriptive outcomes and oocyte-count adjusted odds ratios within each dose-category subgroup.
