# Supplementary material for: Predictive Performance of Oocyte Count for Clinical Pregnancy in GnRH Antagonist IVF Cycles: A Multivariable Analysis of 1171 Fresh Embryo Transfers over a 14-Year Period
Source: Medicina (Kaunas). 2026 Jun 7;62(6):1110. doi: 10.3390/medicina62061110 (PMC13304093; doi:10.3390/medicina62061110)
Supplement: Supplementary file 1 [file medicina-62-01110-s001.zip › Supplementary_Table_S1.pdf]

Supplementary Table S1. Discriminative performance and reclassification analyses comparing oocyte count alone versus the full multivariable logistic regression model.

This supplementary table provides the complete reclassification analysis comparing the full multivariable model (Section 3.3) with oocyte count alone for prediction of the primary composite pregnancy outcome. Estimates are derived from the complete-case multivariable analysis (n = 1,129). Bootstrap confidence intervals were computed from 2,000 non-parametric resamples using the percentile method, with the same random seed used for all bootstrap procedures reported in this manuscript to ensure cross-table reproducibility.

Metric	Estimate	Bootstrap 95% CI	p-value
Discriminative performance (AUC)			
AUC — oocyte count alone	0.532	0.498 – 0.567	—
AUC — full multivariable model	0.564	0.529 – 0.598	—
AUC difference (full − oocyte)	+0.031	−0.007 – +0.068	0.11
Reclassification analyses			
**IDI**	**0.011**	**0.005 – 0.017**	**< 0.001**
**Continuous NRI — total**	**0.135**	**0.014 – 0.251**	**0.03**
Events component	0.058	—	—
Non-events component	0.077	—	—

AUC: area under the receiver operating characteristic curve; CI: confidence interval; IDI: integrated discrimination improvement; NRI: net reclassification improvement.

Interpretation. The IDI of 0.011 indicates that, on average, the full multivariable model yields predicted probabilities that are approximately 1.1 percentage points higher in events than in non-events compared with the oocyte-only model — a difference that is statistically detectable (bootstrap $p < 0.001$) but of modest absolute magnitude. The continuous NRI of 0.135 reflects a small-to-moderate reclassification signal: 5.8% of events are correctly assigned higher predicted probabilities and 7.7% of non-events correctly assigned lower predicted probabilities by the multivariable model relative to oocyte count alone (bootstrap $p = 0.03$). Taken with the non-significant AUC difference ($\Delta = 0.031$, bootstrap $p = 0.11$), these analyses are consistent with the interpretation that the multivariable model provides a statistically detectable but modest incremental discrimination beyond oocyte count alone, and neither approach appears to offer sufficient discriminative performance for clinical decision-making in its current form. These inferences are conditional on the predictors and outcome captured in our dataset; discrimination performance may differ in datasets incorporating embryo morphology, transfer day, or live-birth outcomes.
