# Supplementary material for: Predictive Performance of Oocyte Count for Clinical Pregnancy in GnRH Antagonist IVF Cycles: A Multivariable Analysis of 1171 Fresh Embryo Transfers over a 14-Year Period
Source: Medicina (Kaunas). 2026 Jun 7;62(6):1110. doi: 10.3390/medicina62061110 (PMC13304093; doi:10.3390/medicina62061110)
Supplement: Supplementary file 1 [file medicina-62-01110-s001.zip › Supplementary_Table_S2.pdf]

Supplementary Table S2. Fine-grained stratification of oocyte counts and associated positive-outcome rates in the high-response cohort.

This supplementary table provides a finer stratification of the primary oocyte-count groups used in Table 2 of the main manuscript. The ≥ 16-oocyte group (n = 294 in the main analysis) is further subdivided into 16–20, 21–25, and ≥ 26 oocyte strata to further describe the pregnancy-outcome distribution across the high-response cohort. Five patients with missing oocyte-count data were excluded from the stratification, yielding a total of 1,166 patients. This table is intended as descriptive reference material only; mechanistic interpretations of the observed pattern are not supported by the data presented here and should be informed by prospective designs incorporating embryo-level covariates.

Oocyte count stratum	n	Positive outcome, n	Positive outcome rate (%)	Cryopreservation, n (%)
1–5	213	73	34.3	9 (4.2%)
6–10	376	132	35.1	43 (11.4%)
11–15	283	109	38.5	51 (18.0%)
16–20	146	51	34.9	n.a.
**21–25**	89	46	**51.7**	n.a.
≥ 26	59	19	32.2	n.a.
**Total**	**1,166**	**430**	**36.9**	—

Chi-square test across the six strata: $\chi^2 = 10.63$, $df = 5$, $p = 0.059$.

The six-group comparison reached borderline significance ($p = 0.059$). Descriptively, pregnancy rates were broadly similar across the first five strata (34–39%) with a numerical peak in the 21–25 oocyte stratum (51.7%, n = 89) and a decline in the ≥ 26 stratum (32.2%, n = 59). This descriptive peak should not be interpreted as evidence of an optimal oocyte-count target, as this analysis is unadjusted and the higher-yield strata contain disproportionately younger patients (Table 2 of the main manuscript), which is itself an independent correlate of pregnancy outcome. The pattern is descriptively consistent with the concave trajectory observed in the age- and AFC-adjusted spline regression (Figure 2 of the manuscript), with the caveat that the formal likelihood-ratio test for non-linearity was non-significant ($p = 0.47$). These observations should be interpreted as hypothesis-generating rather than confirmatory, and cumulative outcomes integrating subsequent frozen-embryo transfers from the high-response strata are beyond the scope of the present analysis (see Section 4.1, main manuscript). Cryopreservation data are reported in the main manuscript Table 2 for the four primary groupings; finer stratification of cryopreservation rates within the ≥ 16-oocyte group was not extracted prior to institutional database closure in 2025.

Abbreviations: n.a., not available (sub-stratified cryopreservation data not retrievable after institutional database closure).
