# Supplementary material for: Predictive Performance of Oocyte Count for Clinical Pregnancy in GnRH Antagonist IVF Cycles: A Multivariable Analysis of 1171 Fresh Embryo Transfers over a 14-Year Period
Source: Medicina (Kaunas). 2026 Jun 7;62(6):1110. doi: 10.3390/medicina62061110 (PMC13304093; doi:10.3390/medicina62061110)
Supplement: Supplementary file 1 [file medicina-62-01110-s001.zip › Supplementary_Table_S3.pdf]

**Supplementary Table S3. Missing data summary for variables used in the primary multivariable analysis.**

Missing data were limited (< 1.5% for any individual variable included in the primary model; see table below) and were handled by complete-case analysis, yielding 1,129 patients with complete records (cumulative missingness from 42 patients, 3.59% of the full cohort). The assumption of missing completely at random was considered reasonable given the small volume and clinical context of missingness; no imputation was performed. Reported missingness is derived from the primary institutional dataset as retrieved prior to the 2025 institutional database closure. This supplementary table is provided in accordance with STROBE Item 14(b).

| Variable | Total (n) | Missing (n) | Missing (%) | Used in primary multivariable model |
|---|---|---|---|---|
| Age (years) | 1,171 | 0 | 0.0 | Yes |
| BMI (kg/m²) | 1,171 | 0 | 0.0 | Yes |
| Infertility cause | 1,171 | 0 | 0.0 | Yes |
| Infertility duration (months) | 1,171 | 16 | 1.37 | Yes |
| Basal FSH (IU/L) | 1,171 | 9 | 0.77 | Yes |
| Basal LH (IU/L) | 1,171 | 14 | 1.20 | No (not included) |
| Basal E2 (ng/L) | 1,171 | 7 | 0.60 | Yes |
| AFC | 1,171 | 9 | 0.77 | Yes |
| Stimulation protocol | 1,171 | 0 | 0.0 | Yes |
| Stimulation duration (days) | 1,171 | 1 | 0.09 | Yes |
| Total gonadotropin dose (IU) | 1,171 | 0 | 0.0 | Sensitivity model only |
| Oocytes retrieved | 1,171 | 5 | 0.43 | Yes |
| Embryo transfers | 1,171 | 0 | 0.0 | Yes |
| Primary outcome | 1,171 | 0 | 0.0 | Outcome (dependent) |
| **Complete cases for primary multivariable model** | **1,129** | **42** | **3.59** | — |

*AFC: antral follicle count; AMH: anti-Müllerian hormone; BMI: body mass index; E2: estradiol; FSH: follicle-stimulating hormone; LH: luteinizing hormone. Basal LH was not included in the primary multivariable model because LH status was conveyed by the stimulation regimen dummy variable (rFSH alone vs rFSH + hMG vs hMG vs rFSH + recLH). AMH is not shown in this table as AMH testing was not routinely available throughout the 14-year study period and was therefore not considered for inclusion in the primary model; AFC served as the primary ovarian reserve biomarker.*
