# Supplementary material for: Predictive Performance of Oocyte Count for Clinical Pregnancy in GnRH Antagonist IVF Cycles: A Multivariable Analysis of 1171 Fresh Embryo Transfers over a 14-Year Period
Source: Medicina (Kaunas). 2026 Jun 7;62(6):1110. doi: 10.3390/medicina62061110 (PMC13304093; doi:10.3390/medicina62061110)
Supplement: Supplementary file 1 [file medicina-62-01110-s001.zip › Supplementary_Table_S4.pdf]

Supplementary Table S4. Stimulation-regimen subgroup analysis: descriptive outcomes and oocyte-count adjusted odds ratios within each regimen subgroup.

This supplementary table provides a stimulation-regimen subgroup analysis prepared in response to a peer-review suggestion. Patients were stratified by the stimulation regimen actually administered, and the independent association between oocyte count and a positive primary outcome was examined within each subgroup using a reduced multivariable logistic regression model adjusting for age and antral follicle count (the two principal demographic and ovarian-reserve confounders identified in the primary multivariable analysis, Section 3.3 of the main manuscript). Models were fitted as per-subgroup complete-case analyses; subgroup denominators therefore differ slightly from the overall regimen frequencies reported in Table 1 of the main manuscript.

Stimulation regimen	n (regimen total)	n (analyzable)	Positive outcome, n (%)	Oocyte count aOR (95% CI)	p-value
rFSH alone	626	619	239 (38.6%)	1.007 (0.984–1.032)	0.546
rFSH + hMG	478	475	163 (34.3%)	1.011 (0.976–1.047)	0.555
hMG	61	57	20 (35.1%)	0.933 (0.793–1.097)	0.401
rFSH + recLH	6	—	—	—	—
**Overall (4-group χ^2 for positive-outcome rate)**	**1,171**	**—**	**430 (36.7%)**	**—**	**0.36**

aOR: adjusted odds ratio per additional retrieved oocyte, derived from a per-subgroup multivariable logistic regression model adjusted for age and antral follicle count. CI: confidence interval. The overall 4-group chi-square test for differences in positive-outcome rates across the four stimulation regimens (reported in Table 1 of the main manuscript) yielded p = 0.36, indicating no statistically significant differences in outcome rates between regimens. The rFSH + recLH subgroup (n = 6) was insufficient for a stable multivariable model fit and is presented descriptively only. hMG: human menopausal gonadotropin; recLH: recombinant luteinizing hormone; rFSH: recombinant follicle-stimulating hormone.

Interpretation. Within each of the three regimen subgroups in which a multivariable model could be fitted (rFSH alone, rFSH + hMG, hMG), the adjusted odds ratio for the number of oocytes retrieved was close to 1.0 and not statistically significant (all $p > 0.40$), with 95% confidence intervals consistently encompassing the null. These subgroup findings parallel the primary multivariable analysis (Section 3.3) and indicate that the absence of an independent association between oocyte count and fresh-cycle pregnancy is robust across the principal stimulation-regimen subgroups represented in the cohort. The pattern is also consistent with the unadjusted four-group chi-square test, which showed no statistically significant differences in positive-outcome rates between regimens ($p = 0.36$), suggesting that the choice of stimulation regimen did not by itself confound the principal finding.
