# Supplementary material for: Predictive Performance of Oocyte Count for Clinical Pregnancy in GnRH Antagonist IVF Cycles: A Multivariable Analysis of 1171 Fresh Embryo Transfers over a 14-Year Period
Source: Medicina (Kaunas). 2026 Jun 7;62(6):1110. doi: 10.3390/medicina62061110 (PMC13304093; doi:10.3390/medicina62061110)
Supplement: Supplementary file 1 [file medicina-62-01110-s001.zip › Supplementary_Table_S5.pdf]

Supplementary Table S5. Total gonadotropin dose subgroup analysis: descriptive outcomes and oocyte-count adjusted odds ratios within each dose-category subgroup.

This supplementary table provides a total-gonadotropin-dose subgroup analysis prepared in response to a peer-review suggestion. Patients were stratified into three pre-specified dose categories (< 1,500 IU, 1,500–3,000 IU, and ≥ 3,000 IU) based on the total cumulative gonadotropin dose administered during the stimulation cycle, and the independent association between oocyte count and a positive primary outcome was examined within each subgroup using a reduced multivariable logistic regression model adjusting for age and antral follicle count (the two principal demographic and ovarian-reserve confounders identified in the primary multivariable analysis, Section 3.3 of the main manuscript). Starting-dose categorization was not feasible because the starting dose was not captured as a separate variable in the institutional database; total cumulative dose is therefore used as the dose-exposure metric throughout this analysis. Models were fitted as per-subgroup complete-case analyses; subgroup analyzable sample sizes therefore differ slightly from the overall dose-category frequencies.

Total gonadotropin dose	n (dose category total)	n (analyzable)	Positive outcome, n (%)	Oocyte count aOR (95% CI)	p-value
< 1,500 IU	306	304	119 (39.1%)	1.006 (0.974–1.038)	0.726
1,500–3,000 IU	694	686	256 (37.3%)	1.005 (0.980–1.031)	0.694
≥ 3,000 IU	171	167	50 (29.9%)	0.989 (0.920–1.063)	0.759
**Overall (3-group χ^2 for positive-outcome rate)**	**1,171**	—	**430 (36.7%)**	—	**0.11**

aOR: adjusted odds ratio per additional retrieved oocyte, derived from a per-subgroup multivariable logistic regression model adjusted for age and antral follicle count. CI: confidence interval. The overall 3-group chi-square test for differences in positive-outcome rates across the three dose-category subgroups was non-significant (χ^2 = 4.43, df = 2, p = 0.11). A complementary Cochran–Armitage trend test across the three ordered dose categories yielded borderline evidence of a numerical gradient (z = −1.88, p = 0.061), which is consistent with the previously reported confounding-by-indication pattern in which patients receiving higher cumulative doses tend to have lower ovarian reserve and other unfavourable baseline characteristics; this descriptive gradient is fully attenuated after multivariable adjustment, as already demonstrated in the dose-inclusive sensitivity model (Section 3.3 of the main manuscript). IU: international units.

Interpretation. Within each of the three pre-specified total-dose subgroups, the adjusted odds ratio for the number of oocytes retrieved was close to 1.0 and not statistically significant (all p > 0.65), with 95% confidence intervals consistently encompassing the null. These subgroup findings parallel the primary multivariable analysis (Section 3.3) and indicate that the absence of an independent association between oocyte count and fresh-cycle pregnancy is robust across the principal total-dose subgroups represented in the cohort. The 3-group chi-square test for raw positive-outcome rates was non-significant (p = 0.11), and the borderline numerical trend across ordered dose categories (Cochran–Armitage p = 0.061) is consistent with confounding by clinical indication—higher cumulative doses are administered to patients with lower ovarian reserve and other unfavourable baseline characteristics—and is fully attenuated after multivariable adjustment, in agreement with the dose-inclusive sensitivity model already presented in the main manuscript.
